# Advancements in cathode catalyst and cathode layer design for proton exchange membrane fuel cells

**DOI:** 10.1038/s41467-021-25911-x

**Published:** 2021-10-13

**Authors:** Yanyan Sun, Shlomi Polani, Fang Luo, Sebastian Ott, Peter Strasser, Fabio Dionigi

**Affiliations:** 1 The Electrochemical Energy, Catalysis, and Materials Science Laboratory, Department of Chemistry, Chemical Engineering Division, Technical University Berlin, Straße des 17. Juni 124, 10623 Berlin, Germany; 2 School of Materials Science and Engineering, Central South University, 410083 Changsha, Hunan China

**Keywords:** Electrocatalysis, Energy, Devices for energy harvesting, Fuel cells

## Abstract

Proton exchange membrane fuel cells have been recently developed at an increasing pace as clean energy conversion devices for stationary and transport sector applications. High platinum cathode loadings contribute significantly to costs. This is why improved catalyst and support materials as well as catalyst layer design are critically needed. Recent advances in nanotechnologies and material sciences have led to the discoveries of several highly promising families of materials. These include platinum-based alloys with shape-selected nanostructures, platinum-group-metal-free catalysts such as metal-nitrogen-doped carbon materials and modification of the carbon support to control surface properties and ionomer/catalyst interactions. Furthermore, the development of advanced characterization techniques allows a deeper understanding of the catalyst evolution under different conditions. This review focuses on all these recent developments and it closes with a discussion of future research directions in the field.

## Abstract (web summary)

The high platinum loadings at the cathodes of proton exchange membrane fuel cells significantly contribute to the cost of these clean energy conversion devices. Here, the authors critically review and discuss recent developments on low- and non-platinum-based cathode catalysts and catalyst layers.

## Introduction

Increasing global energy demand and environmental concerns have driven the development of sustainable energy conversion and storage technologies. Among them, proton exchange membrane fuel cells (PEMFCs) stand out owing to their unique advantages of zero-emission of greenhouse gases, high theoretical power density, and energy conversion efficiency. Moreover, PEMFCs find applications in the fields of transportation, material handling, stationary, and portable power generation. In principle, PEMFCs can directly convert renewable chemical energy into electrical energy based on two key electrochemical reactions: the hydrogen oxidation reaction (HOR) at the anode and the oxygen reduction (ORR) at the cathode. Relative to the HOR, the sluggish kinetics and high over-potential of the ORR is the critical technical bottleneck for the overall performance of PEMFCs. Currently, the state-of-the-art (SoA) ORR catalysts in PEMFCs consist of Pt-based nanoparticles (NPs), and have a broad deployment in hydrogen-powered fuel cell electric vehicles (FCEVs). However, a significant amount of Pt is required to achieve high voltage output and sufficient power densities, which accounts for a substantial part of the total cost of the PEMFCs stack^1^. Therefore, in the last two decades, research has focused on reducing or even eliminating the amount of Pt in cathodes by developing catalysts based on low usage of platinum group metals (PGMs) and alternative PGM-free catalysts^2^.

To realize low-Pt-loaded PEMFC electrodes maintaining high performance at high current densities, the local O_2_ depletion must be avoided at the triple-phase boundary, where the ORR-active metal sites on the support are in contact with oxygen molecules, water generated at the sites, and the proton-conducting medium (the ionomer or the water). Thus, the development of higher active and stable PGM-based catalysts must be combined with the engineering of the triple-phase boundary microenvironment to achieve superior power density. Alternatively, nanostructured metal–nitrogen–carbon (M–N–C, M = Fe, Co, Ni, Mn, Cu, Sn, etc.) materials have been widely considered the most promising PGM-free ORR catalysts owing to their encouraging catalytic activity^3–15^. The ideal M–N–C ORR catalysts in the term of power generation should catalyze directly the four-electron ORR to achieve the highest energy conversion efficiency. Charge transfer and mass transport properties of the cathode catalyst layer (CL) in membrane electrode assemblies (MEA) strongly affect the effective utilization of the catalytic active sites in M–N–C catalysts. In particular, high loading of M–N–C catalysts is needed to achieve sufficient activity, making the cathode layers of M–N–C catalysts much thicker than that of PGM-based materials. This point results in inferior mass transport and unsatisfactory interfacial charge transfer. Besides, the level of technological maturity of this approach is lower relative to the PGM-based systems in addition to the challenging stability.

**Fig. 1:**
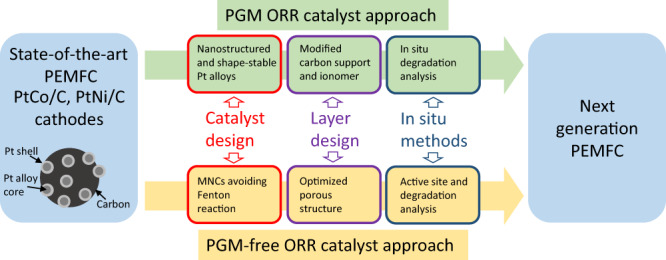
Scheme of the topics discussed in the review. The scheme shows two alternative routes to bring PEMFC performance to the next performance generation. The two strategies employ either PGM-based or PGM-free cathode catalysts, respectively, and involve catalyst design, layer design, and the development of in-situ analysis methods. Promising catalyst designs are indicated, as well as important key aspects addressed by layer designs and in-situ methods.

In addition to these two main research directions, composite catalyst systems comprising PGM-free and PGM-based catalysts were also investigated^16^. To discover novel materials, the combination of rotating (ring-)disk electrode (RRDE/RDE) screenings and theoretical screening methods has been proven to be a powerful approach. Recent advances have been achieved also thanks to collaborative efforts in large research consortia between industries, research centers, and universities, i.e. EU/FCH JU-lead efforts (GAIA, CRESCENDO, PEGASUS) in Europe, ElectroCat in the United States (US), etc. Particularly, MEA-based fuel cells (FCs) tests demonstrated power density at high current loads meeting the upcoming commercialization cost targets of the automotive and transport sector. This review focuses on recent relevant developments and important advances with respect to the catalyst and support materials for the cathode layer design of PEMFCs (Fig. 1), and future research directions and perspectives in this field are also discussed.

## Low-PGM electrocatalysts and triple-phase boundary design

### Design of low-PGM catalysts

Several strategies have been developed to improve the activity and stability of PGM-based catalysts, as summarized in Table 1.

**Table 1:** Strategies to improve the activity and stability of PGM-based catalysts.

Design strategy	Targeting enhancement in	Selected examples
Alloy Pt with 3*d* transition metals, dealloyed core-shell catalysts	Activity	PtNi^18,36,116^, PtCo^69^
Alloy Pt with lanthanides and rare earths	Activity (and stability)	Pt–Gd alloy^19^, Pt–Y alloys^117^
Shaped alloy nanoparticles and nanostructures	Activity	oh-PtNi^21–23,118^, PtNi nanocage^24^, PtNi nanoframes^26^
Ordered intermetallic nanoparticles	Activity (and stability)	Intermetallic L1_0_–CoPt/Pt^28^
Second coordination shell effects on Pt sites with optimized coordination	Activity	Defective Pt (111)^31^
Intrinsic strained nanosheets	Activity	Ultrathin Pd nanosheets^30^
Conformal ultrathin Pt shell	Stability	On oh-PtNi^32^, on carbides cores^34^, on Pd_*x*_Au alloy^119^
Surface decoration with Au clusters	Stability	On Pt NPs^35^
Thermal annealing/leaching treatments	Stability (and activity)	PtCo NWs^27^, oh-PtNi^38^
Surface doping	Stability (and activity)	Surface doped oh-PtNi with Mo^23^, Rh^41^

The main targeted performance to improve is indicated, together with selected examples, which are discussed in the text.

In principle, tuning the oxygen adsorption energy by adjusting the compressive strain of the Pt surface of Pt catalysts is a useful approach to improve the ORR activity. In bimetallic PtM catalysts, alloying Pt with lanthanides or 3*d* transition metals, results in a volcano-like relationship between the catalytic activity and the electronic structure (*d*-band center energy)^17,18,19^. Since most 3*d* transition metals are smaller than Pt, their incorporation into face-centered cubic Pt causes, after electrochemical dealloying, compressive strain in the Pt enriched shell (core@shell catalysts) that lowers the adsorption energy of the oxygen-containing intermediate species.^17,20^ Similarly, the introduction of the larger lanthanides can also induce the rearrangement of surface Pt atoms in the so-called Kagome layer thus generating compressive strain^19^. For Pt alloys, Stamenkovic et al. reported the record high activity enhancement of Pt_3_Ni (111) single crystals with a thin strained Pt shell^18^. Transferring these findings on single crystal surfaces to the nanoscale guided the fabrication of active catalysts with exposed Pt (111) facet. Various PtM morphologies including PtNi octahedra (oh-PtNi)^21,22,23^, nanocages^24,25^ and nanoframes^26^, PtCo nanowires (NWs)^27^, L1_0_-CoPt intermetallic catalysts with a thin strained Pt shell^28^, and PtPb@Pt nanoplates exposing the stable Pt (110) facet were also fabricated^29^. In the work with PtPb@Pt nanoplates, the tensile strain produced on Pt (111) facet was reported to induce stronger oxygen binding to Pt surface atoms. Another reported strain type originated from a few intrinsic atomic layers of ultrathin metal sheets rather than underneath foreign metals^30^.  Finally, a strategy to improve the activity in the case of non-alloyed metallic catalysts was also shown by Calle-Vallejo et al. by using a generalized coordination number concept^31^. Based on this concept a highly active defective Pt (111) was designed.

Besides the enhancement of the ORR activity, improving the durability is also a key research challenge. Conformally deposited ultrathin Pt shells as an approach to improve durability have mostly been investigated^32,33^. The ligand effect, typically effective over one to three Pt atomic layers, arises from the proximity of transition metals with different electronegativity. This provides a direct electron interaction, weakening the adsorption of the oxygen-containing intermediate species. Therefore, the thickness of the Pt shell is very important. For instance, the oh-PtNi@Pt_1.5ML_ exhibited improved durability compromising mass activity (MA) only by 13% relative to the oh-PtNi catalyst^32^. Göhl et al. ^34^ showed that atomically thin Pt shells stabilize titanium tungsten carbide cores as well, even at strong oxidizing potentials. Adzic and co-workers reported the deposition of a Pt monolayer on Pd NPs to improve long-term stability in fuel cells^33^. Besides, Pt cathode electrocatalysts can be stabilized against dissolution under potential cycling regimes by modifying Pt nanoparticles with Au clusters to raise the oxidation potential of Pt^35^. Thermal annealing of PtM alloys alone or combined with acid leaching/dealloying is commonly used to achieve the desired surface structure and stable composition profile^36^. For example, Li et al. ^37^ reported the synthesis of jagged Pt NWs (J-PtNWs) with the unusually high, yet to be reproduced, MA of 13.6 A mg_Pt_^–1^ through the following three steps: solution-synthesized Pt/NiO core@shell NWs, thermal annealing, and electrochemical dealloying. For anisotropic structures such as J-PtNWs, the high activity originates from stressed and undercoordinated Pt surface atoms. Interestingly, in the case of octahedral nanoparticles, an annealed oh-PtNi at 300 °C (oh-PtNi-300) in a reducing atmosphere exhibited increased activity whereas decreased activity was reported for annealing at 500°C^38^. The microstrain developed in the different heating steps proved to be the best descriptor of the activity shifts^39^.

### Alloying oh-PtNi catalysts with a ternary metal

**Fig. 2:**
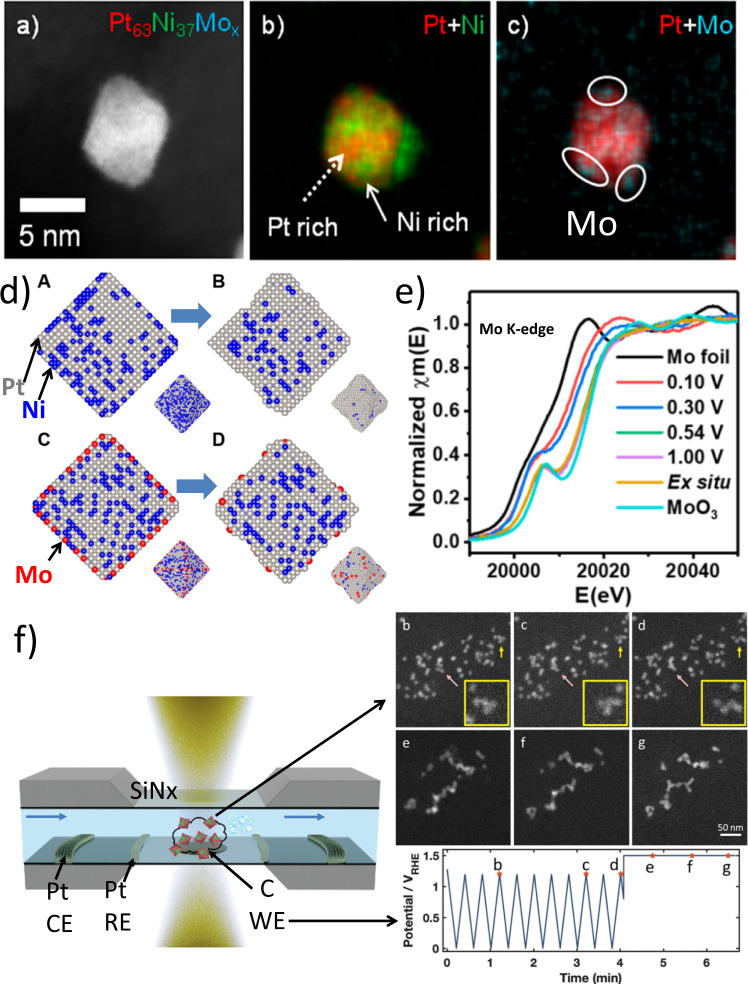
Enhancing octahedral PtNi NPs catalysts: understanding surface doping and degradation mechanisms. **a**–**c** TEM images showing elemental anisotropic Pt and Ni distribution and Mo surface dopants location on an oh-PtNi(Mo) catalyst^44^. **d** Middle cross-section of PtNi before (A) and after (B) and PtNiMo before (C) and after (D) evolution under kinetic Montecarlo (KMC) simulations at a temperature of 27 °C. The insets show snapshots of the 3D particles^42^. **e** The Mo K-edge XANES spectra were collected ex situ and in situ in an O_2_-purged 0.1 M HClO_4_ electrolyte as a function of applied potentials^42^. **f** Electrochemical liquid cell for in-situ TEM showing an example of degradation pathway involving coalescence (pink arrows) and particle motion (yellow arrows) during an electrochemical protocol. The potential profile over time is labeled with marked points (**b**–**g**) corresponding to the images. Right image-side adapted from ref. ^47^. Panels **a**–**c** from ref. ^44^ under CC BY NC licence. Panels **d** and **e** adapted with permission from ref. ^42^. Copyright 2017 American Chemical Society.

oh-PtNi catalysts typically produce enhancement activity factor respect to Pt order of magnitude lower than the Pt_3_Ni (111) single crystal record activity and suffer from poor stability due to Ni leaching under electrochemical conditions^21,40^. Huang’s group investigated systematically the third metal as a surface dopant to further improve the performance of oh-PtNi^23^, through the optimization of local oxygen binding energies and stabilization of Ni in Pt lattices^41–43^. Further, computational results (Fig. 2a–e) by the Mueller group demonstrated that the surface presence of Mo as oxides induces the increase of subsurface Ni and stabilizes undercoordinated Pt sites^42^. Later, X-ray fluorescence spectroscopy and in-situ X-ray scattering experiments proved that the high durability of oh-PtNi(Mo) catalysts originated from better Ni retention^44^. Besides, Beermann et al.^41^ reported on the uniquely enhanced activity and stability of Rh-doped oh-PtNi catalysts compared to the pristine oh-PtNi catalyst. In particular, the octahedral shape was almost unaffected even after 30k cycles between 0.05 and 1.0 V_RHE_. The enhanced stability was attributed to the suppression of Pt migration at the surface rather than Ni dissolution.

### In-situ TEM and other advanced-characterization-techniques of shaped catalysts

In-situ characterization techniques such as in-situ X-ray techniques (absorption spectroscopy^42^, diffraction, small and wide-angle scattering)^38,45^, Fourier-transform infrared (FTIR) spectroscopy^38^, TEM^46^, and scanning flow cell coupled with mass spectrometry^34,47^ are widely employed to study the degradation mechanism of catalysts. For shaped catalysts, the degradation of the facets that give the catalyst its superior activity inhibits their use in fuel cell devices. The electrochemical liquid cell in-situ TEM (Fig. 2f), provides an accurate correlation between the applied electrochemical potential and the microstructural response of the catalyst. Nevertheless, the thick SiN_*x*_ membrane (20–50 nm), the electrolyte decomposition, and thickness set limitations on the radial resolution. Beermann et al.^46^, reported on carbon corrosion, particle movement, and particle coalescence as the main microstructural responses to potential sweeps and holds in regimes where carbon corrosion occurs. During an extremely high potential excursion, the shaped NPs on the carbon support became mobile and agglomerated facet-to-facet within 10 s. In-situ annealing TEM monitors time-resolved changes in catalyst surfaces and morphology at different temperatures and atmospheres^48^. Coupling in-situ thermal annealing TEM with in-situ X-ray diffraction experiments allows tracking the morphological and structural changes during annealing steps^38^. Shviro and Gocyla^49^ followed changes in oh-PtNi catalysts from a segregated structure to an alloyed shell configuration to a quasi-spherical structure as a function of temperature under reducing conditions. Exposure to an oxidizing environment leads to the oxidation of the carbon support and the formation of cubic NPs due to the formation of CO before it is finally oxidized to CO_2_. In addition, Xiong and Yang recently published a comprehensive quantitative study of the dynamic order–disorder phase transition of binary intermetallic Pt_3_Co NPs during post-synthesis annealing using in-situ synchrotron XRD and in-situ STEM^50^.

Besides in-situ TEM, the development of new techniques is needed to study the local non-uniform degradation of Pt catalysts on a large electrode scale. In this regard, Cheng et al. ^51^ employed synchrotron X-ray microdiffraction to spatially resolve and quantify the degradation of Pt catalyst by mapping the size of Pt particle. In addition, direct evidence of liquid water accumulation at the anode leading to severe ionomer swelling, performance loss, and cell drying due to undesirably low water content in the cathode was obtained using operando neutron imaging and operando micro-X-ray-computed tomography^52,53^.

### Translating high RDE activities to MEA-based fuel cells for PtM alloys

**Fig. 3:**
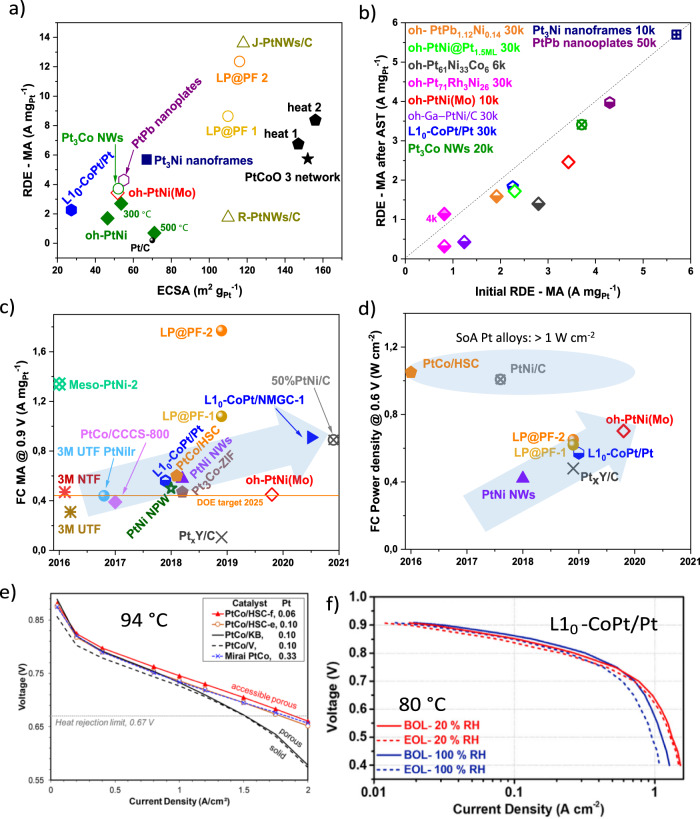
Current performance of PGM catalysts. **a** MAs at 0.9 V_RHE_ as a function of ECSAs, and **b** MAs at 0.9 V_RHE_ before and after a different number of potential cycles, from RDE measurements. In panel (**a**) the ECSA was obtained from either hydrogen underpotential deposition (H_upd_, open symbols) or CO stripping (full colored symbols). **c** MAs at 0.9 V for low-PGM catalysts calculated from PEMFC performance with H_2_–O_2_, 1.0 bar, 80 °C, and 100 RH %, and **d** power density performance of promising low-PGM catalysts evaluated at 0.6 V in PEMFCs under hydrogen/air feeds at different loadings and conditions: 100% RH, 80 °C, stoichiometries are H_2_/Air 1.5/2 and absolute pressure of: For Pt_*x*_Y/C: 170 kPa_abs_. For PtNi/C and oh-PtNi(Mo): 100 kPa_g, inlet_. For the remaining catalysts: 150 kPa_abs_. While SoA Pt alloys such as PtCo and PtNi show the highest power densities, promising catalysts with alternative designs have improved their performances over the recent years, as highlighted by the arrow in panel (**d**). Catalysts include Pt_*x*_Y/C^117^, PtNi NWs^120^, nanoframes^26^ and nanopinwheels (NPWs)^121^, Pt_3_Co NWs^28^, PtCo/HSC^69,122^, L1_0_-CoPt^27^, L1_0_-CoPt on hydrogel (L1_0_-CoPt/NMGC-1)^57^, 50% PtNi/C^56^, PtNi/C^123^, PtPb nanoplates^29^, oh-PtNi@Pt_1.5ML_^32^, R-PtNWs and J-PtNWs^37^, Oh-PtNi (300 and 500)^38^, LP@PF^17^, PtCoO 3, heat 1, heat 2^74^, Oh-PtNi(M), M = Mo^23,44^, Rh^41^, Ga^43^, Co^124^^,^ and Pb^125^. 3M UTF PtNiIr^126^, Meso-PtNi-2^127^, 3M UTF and NTF^126^, PtCo/CCCS-800^58^ and Pt_3_Co-ZIF^16^. Panel **b** and **c** are adapted from ref. ^2^. **e** Air/H_2_ fuel cell performance of SoA PtCo catalysts on different carbons. Cathode Pt loadings in the legend, 0.25 mg_Pt_ cm^−2^ for the anode. Anode/cathode operating conditions: H_2_/air, 94 °C, 65/65%RH, 250/250 kPa_abs,outlet_, stoichiometries of 1.5/2, 50 cm^2^ active area^69^. **f** H_2_/air fuel cell polarization curves obtained with L1_0_-CoPt/Pt catalysts, showing SoA mass activity in H_2_/O_2_ feed above 2025 US Department of Energy (DOE) target (cf. panel **b**). Conditions: 8 wt% Pt content, Pt loading of 0.105 mg_Pt_ cm^−2^, 80 °C, RH in the legend, 150 kPa_abs_ H_2_/air, gas flow rate of 500/1000 sccm^27^. Panel **e** adapted with permission from ref. ^69^. Copyright 2018 American Chemical Society.

While FCEVs have been commercialized, the use of the most advanced electrocatalysts for ORR that were individuated by RDE screenings (Fig. 3a and b) has not yet been met due to the insufficient activity and durability in MEA-based FCs^54^. The promising RDE-level ORR performance does not always ensure the conversion of the activities into real MEA-based FCs. Typically, a performance drop of more than three times is observed (Fig. 3c). In the case of oh-PtNi(Mo)^44^, a remarkable RDE-level MA of 3.4 A mg_Pt_^−1^@0.9 V_RHE_ was translated to an MEA-based single-cell MA of 0.45 A mg_Pt_^−1^ at 0.9 V, indicating a lower enhancement factor in MEA-based FCs tests than in RDE tests even though met the DOE 2025 milestone (0.44 A mg_Pt_^−1^)^55^. Particularly remarkable is the recently reported MA of 0.89 A mg_Pt_^−1^ at 0.9 V for 50 Pt wt%-PtNi catalyst by Johnson Matthey Fuel Cells within the GAIA framework^56^. Similar activity, slightly surpassing 0.9 A mg_Pt_^−1^ at 0.9 V, was also recently reported for L1_0_-CoPt catalyst on hydrogel-derived carbon support by the Spendelow group^57^. In addition, the use of metal-organic frameworks (MOFs) as templates for carbon structures, especially carbon supports derived from zeolitic imidazolate frameworks (ZIFs), has shown great promise^16^. In a related study, Co NPs encapsulated in an N-doped graphitic carbon shell diffused under the polymer protective layer to the Pt NPs surface and formed an active, chemically ordered, face-centered tetragonal PtCo/CCCS catalyst^58^. The synergistic catalysis between strained Pt shells over a PGM-free catalyst substrate resulted in excellent fuel cell performance. The difference of the performance of RDE and MEA-based FCs tests highlights the necessity of the development of MEA in the early stages of catalyst research and development. This would be also important to improve the power density performance in fuel cells running under H_2_/air feeds (Fig. 3d). New synthetic approaches to develop active and stable Pt alloy catalysts embedded in a high surface area carbon (HSC) support were developed for low PGM cathode catalyst, achieving new SoA H_2_/Air performance in PEMFC (Fig. 3e). These catalysts achieved power densities at 0.6 V above 1 W cm^−2^, which are not yet reached by other catalysts showing SoA MA (Fig. 3c, d, f). In general, the reported high mass activities of PGM-based catalysts in PEMFCs were obtained under wet humidity and at low cathode loadings (i.e. <0.10 mg_Pt_ cm^−2^), where achieving high power densities is very challenging. To the best of our knowledge, such high activities have not been reported at higher loadings, i.e. 0.2 mg_Pt_ cm^−2^. It seems that the challenges in translating the performance from RDE to MEA are associated with the different testing conditions, in terms of proton/oxygen transport and temperatures, as well as to the preparation of the MEAs, which is often not optimized for the new advanced design catalysts, but for smaller Pt and Pt dealloy/alloy NPs. Therefore, we believe that the interaction with the ionomer and the carbon plays a key role in this issue. Future directions to translate the performance from RDE to MEA might involve the combined effort in improving both the PtM catalyst and the support design. This aspect and more in detail the design of the triple-phase boundary interface will be discussed in the following section.

### Nanoscale engineering of the triple-phase boundary

The ionomer plays a key role in the CL as a binding and proton-conducting material. However, at the triple-phase boundary, direct contact of the ionomer side chains with the active catalyst sites leads to a detrimental poisoning effect by the electronegative functional groups of the ionomer (–SO_3_^−^, –O^−^)^59,60^. Takeshita et al. studied the ionomer poisoning effect by CO stripping under fluorocarbon-fluid-filled conditions and determined a reduction in MA up to a factor of 5 by direct contact of the ionomer with the active sites^60^. To reduce this poisoning effect, Yamada et al. deposited a thin carbon layer (<1 nm) on the metal particles by a post-thermal dopamine treatment^61^. This in turn reduced the contact of the active sites with the ionomer, thus suppressing sulfonate adsorption, while still ensuring sufficient proton conductivity throughout the CL^61^. As an alternative approach, Kodama et al. investigated the configuration of ionomers containing side chains of different lengths with or without an additional ether group in the midpoint of the side chain^59^. Both the terminal sulfonate and the ether group were found to tend to block the active sites via the oxygen atoms. Moreover, long side chains were found to effectively block the Pt surface due to their flexibility. In contrast, short side chains are less flexible and develop a strain on the ionomer backbone (main chain) upon adsorption. Due to the consequential ionomer deformation, less sulfonate adsorption is observed for ionomers with short side chains^59^. Nonetheless, a translation of these RDE findings into MEA, resulting in a correlation between ionomer’s SO_3_^−^ group coverage and MA, is still missing.

Besides poisoning effects, short side chains and the microporous structure of the CL can also negatively affect the mass transport at high current density, due to a parallel alignment of the ionomer chains’ PTFE backbone towards the catalysts’ surface^62^. Harada et al. provided the first structural evidence of local oxygen diffusion hindrance at the triple-phase boundary^63^. They showed that a denser, water-depleting ionomer layer of 3 nm lies on top of the metal particles and leads to an activation barrier for oxygen diffusion from the gas phase to the active site. To minimize this diffusion barrier effect, Modestino and coworkers created a new group of porous, proton-conducting ionomers based on a perfluoro(2-methylene-4-methyl-1,3-dioxolane) (PFMMD) backbone structure enabling high oxygen permeability^64^. With a different approach, Yoshino et al. presented an electrospinning method manufacturing CL with ionomer nanofibers^65^. They achieved reduced oxygen transport resistances and postulated a decrease in ionomer density near the active sites. Another approach is based on the coulombic interaction of the negatively charged side chains with positively charged dopants in the support matrix. Orfanidi et al. and Ott et al. presented a tailoring method with N-doping of the carbon surface to achieve a more homogeneous ionomer distribution over the entire catalyst surface. This in turn resulted in a thinner, uniformly connected ionomer layer that reduced oxygen diffusion resistance through the pore structure and through the ionomer itself^66,67^.

The interaction between catalyst and ionomer is also affected by the pore structure of the carbon support and the precipitation method of metal, which define the location of the active sites. These can be exposed on the outer or inner surface of the support, and their ratio can be determined electrochemically by a humidity-dependent CO stripping method^68^. To achieve a high current density with lower mass transport, exposing metal particles on the outer surface is favorable due to the easier accessibility by the reactant gas, whereas additional transport barriers need to be overcome on the inner surface. However, such exterior particles in closer contact with the ionomer exert a stronger poisoning effect, reducing the MA. Therefore, it would be desirable to have the catalyst particles inside specifically designed pores that will not allow the penetration of ionomer in the size of 10 nm, while maintaining adequate oxygen transport. Padgett et al. presented a detailed comparative study of internally and externally surface-located catalyst particles investigating differences in particle size and degradation mechanisms^68^. The internally deposited particles were accessible via bottleneck pores, which are known to increase mass transport issues, but revealed smaller particle sizes after the degradation tests and therefore higher electrochemical surface areas (ECSA). Furthermore, they suffered less degradation as particle coalescence is minimized by the larger interparticle distance that is induced by the pore structure. Yarlagadda et al. recently presented a synthetic approach to overcome this trade-off by creating accessible pores in the 4–7 nm range, thereby reducing the bottleneck negative effect: particles in these pores do not suffer from direct ionomer poisoning and minimize the oxygen transport resistance^69^. This improves the kinetic and transport limitation regimes during cell operation. Based on this approach, Ramaswamy et al. investigated the influence of micropores (<2 nm) and macropores (defined here as >8 nm) on the cell performance limitations^70^. The authors showed a clear correlation of the micropore volume of the supports with the local oxygen resistance and of the macropore volume with the proton resistance. Micropores act as a bottleneck to oxygen transport to the active sites and therefore a low micropore volume is preferable. In addition, an increased macropore volume is associated with an increased macro-surface area and leads to a more interrupted ionomer layer, which increases proton transport resistance^70^. Therefore, macropore volume should be also minimized.

**Fig. 4:**
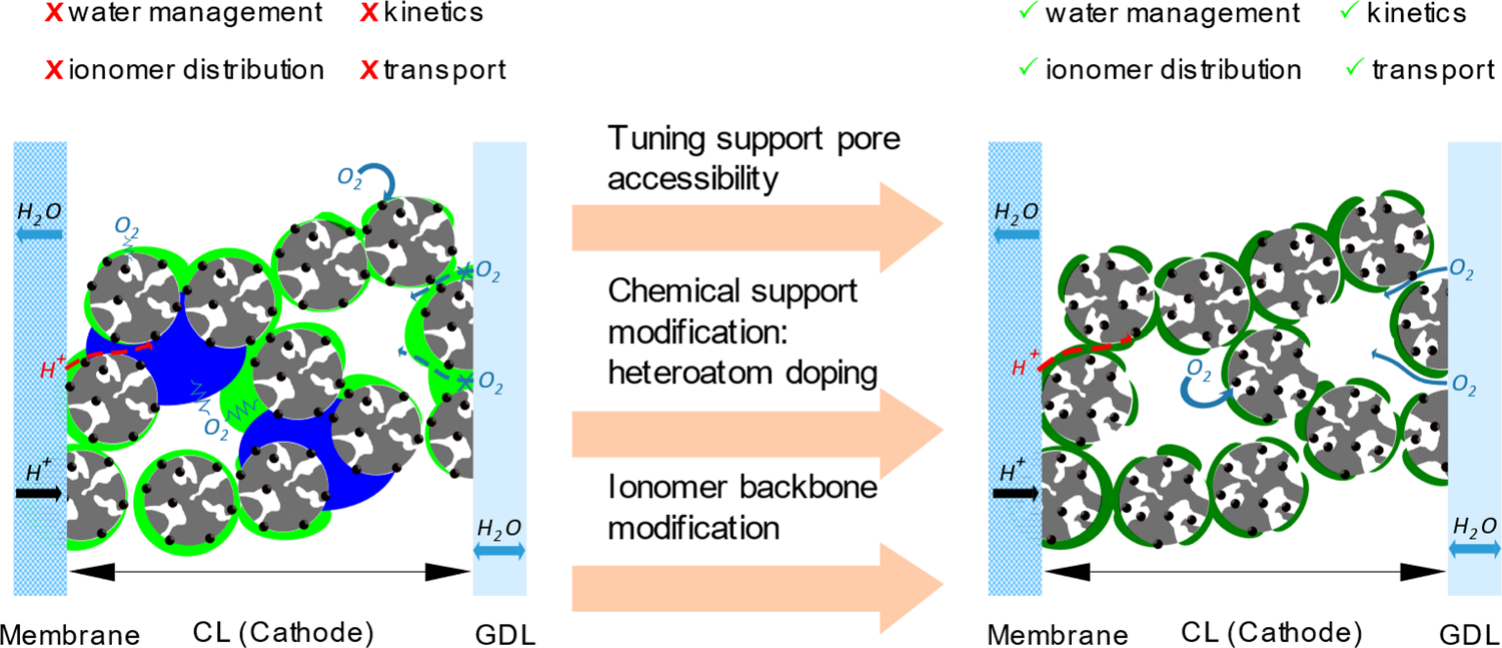
Catalyst layer optimization. Schematic illustration of a catalyst-coated membrane/gas diffusion layer (CCM/GDL) set consisting of a conventional high surface area carbon (gray porous sphere)-supported catalyst layer (left) and a tuned catalyst layer revealing optimized support/ionomer configuration (right). Proton and oxygen pathways through the ionomer layer (green) and the water droplets (blue) reaching the active sites (black sphere) are illustrated with red and blue arrows, respectively, and marked for high resistance pathways using the conventional sign of resistance. Red and green checkmarks indicate aspects that are challenging in current cathodes and are expected to be addressed in future catalyst layers. Three modification approaches (pink arrows) leading to improve catalyst layers are highlighted in the center of the illustration.

To properly tackle water management in these structured electrodes, the porosity of the CL and its thickness must be considered. Sassin et al. evaluated CL with different thicknesses according to their porosity and mass transfer properties^71^. The authors reported the pore size distribution within the CL to be independent of the layer thickness, but the porosity decreased with the electrode thickness due to a more densely packed layer. Furthermore, the low porosity associated with the denser layers hinders oxygen mass transport to the active site, while a high porosity in a thin CL favors flooding, which, in turn, increases oxygen transport resistance as well. Based on these studies we believe that to realize low PGM loadings, a carbon with high surface area, open-pore structure, and accessible pores is desired. To achieve this type of support material, a physically fine-tuned carbon synthesis procedure might be combined with a chemical support modification approach, as illustrated in Fig. 4.

In addition to the concept of the carbon-supported catalyst, various attempts have been made to dispense with the use of carbon as support material but maintaining a porous structure to allow sufficient mass transport^72^. Snyder et al. synthesized a nanoporous core–shell-like Ni–Pt alloy and impregnated it with an ionic liquid that provides high oxygen solubility^73^. Nevertheless, no conclusions can yet be drawn for such a catalyst in terms of operation in a PEMFC stack and water management issues. Another example of self-supporting catalysts was reported by Sievers et al.^74^. In their work nanostructured Pt–CoO networks were obtained by alternating Co and Pt magnetron sputtering, reducing the influence of the ionomer on the proton conductivity since the proton conductivity appeared to proceed via the catalyst surface itself.

An alternative promising approach for reducing poisoning effects of active sites is the addition of adjuvants or ionic modifiers. The use of ionic liquids as additives enable a local surrounding of the active sites with high oxygen solubility, creating a protective layer to prevent surface adsorption of sulfate groups and carbon corrosion^47^. Notably, this layer still ensures sufficient ORR since it does not affect the overall mechanism. Here melamine shall be pointed out as one promising additive, as shown in a recent work from Zorko et al.^75^. To design an improved triple-phase boundary, one should start optimizing the ink composition, since it influences the ionomer distribution^76^. Additionally, the use of a porous backbone-based ionomer will improve oxygen permeability whereas additives such as ionic liquids reduce poisoning issues. A physical and chemical support modification might enable sufficient mass transport properties of the CL.

## PGM-free electrocatalysts and layers as a promising alternative

Nanostructured M–N–C materials, especially Fe–N–C materials, have been widely investigated as the representative of PGM-free ORR catalysts due to their appealing catalytic activity^1,77–82^. During the preparation process, different Fe-containing species could be simultaneously formed, including atomically dispersed Fe–N_*x*_ moieties and iron carbide embedded into nitrogen-doped graphitic carbon layers (Fe_3_C@NC), accompanying the generation of nitrogen functionalities moieties within the carbon matrix (NC). To elucidate the nature of catalytically active sites, various Fe-specific and surface-selective spectroscopic techniques have been explored, including Mössbauer spectroscopy, X-ray absorption spectroscopy (XAS), and nuclear resonance vibrational spectroscopy (NRVS)^83,84^. Meanwhile, various molecule probes have been also explored to identify the specific ORR-active sites through the selective binding to certain chemical moieties, including NO^83^, CO, PO_4_^3−^ ^85^, NO_2_^−^^86^ and R-SO_3_^−^^87^. For example, NO has been applied to selectively detect gas-accessible reactive Fe sites toward the ORR through the formation of stable Fe−NO adducts^83^.

**Fig. 5:**
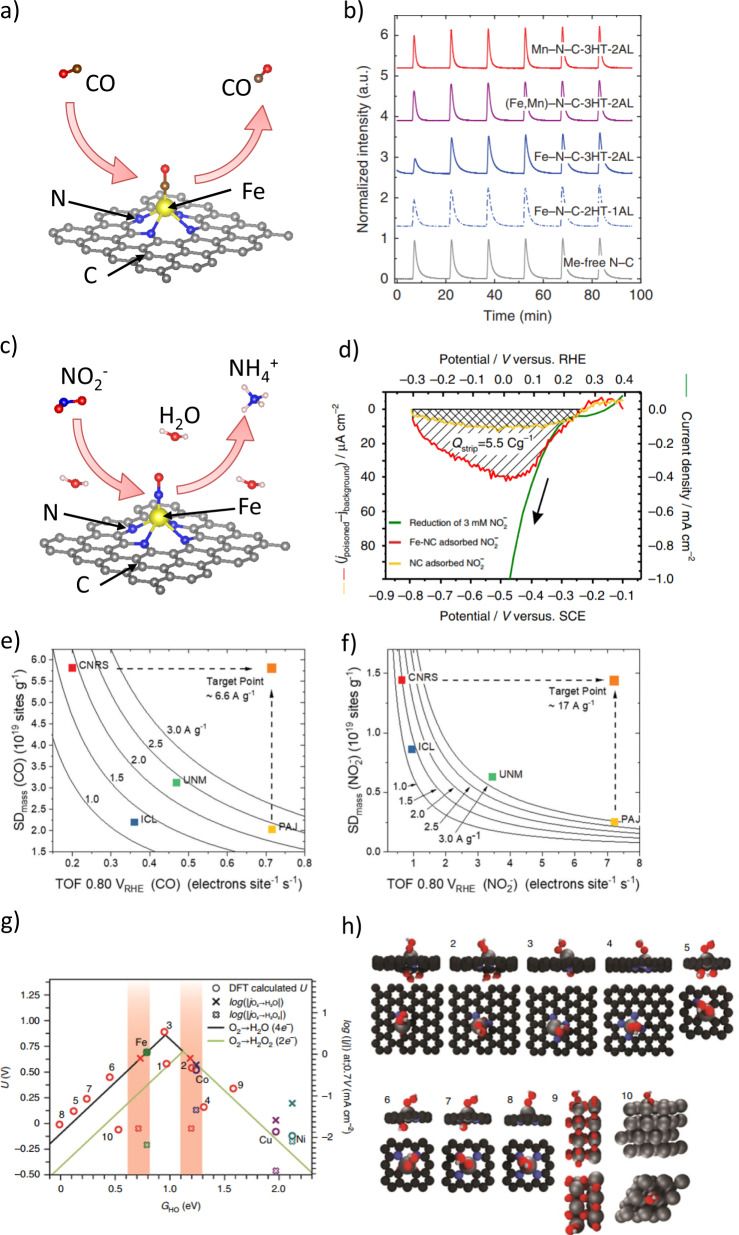
Surface density and structure of the ORR active sites in MNCs. **a** Scheme depicting CO cryo adsorption and temperature-programmed desorption (TPD), which enable counting the catalytically active FeN_*x*_ sites on the surface of previously thermally cleaned Fe–N–Cs^88^. **b** CO pulse chemisorption profiles of MNCs catalysts: N–C (gray trace), two differently treated Fe–N–Cs (dashed and solid blue traces), (Fe,Mn)–N–C (violet trace), Mn–N–C (red trace)^89^. **c** Scheme for the nitrite reduction stripping charge method, showing the adsorption and stripping processes which enable calculating the SD on Fe–N–C under in-situ electrolyte conditions. **d** Comparison between homogeneous reduction of aqueous nitrite (green curve), and excess current associated with reductive stripping of nitrite adsorbed intermediates on Fe–N–C (red) or N–C catalyst (yellow). The reductive stripping curve is produced by subtracting the potential scan curves in N_2_-saturated electrolyte for unpoisoned and nitrite poisoned catalyst, where *Q*_strip_ is the coulometric charge associated with the NO stripping peak^90^. (**e**-**f**) Site density-ORR turnover frequency (SD-ORR TOF) maps were obtained by plotting the Fe surface SD and the corresponding TOF for four Fe–N–Cs catalysts with iso-mass activity hyperbolic curves at 0.80 V_RHE_. SD derived from CO (**e**) and NO_2_^−^ (**f**) adsorption/desorption methods. Dashed arrows indicate examples of catalyst target performance^86^. **g** ORR activity volcano plot for SnNC structures and other MNCs (M = Cu, Co, Ni and Fe) displaying computed onset potentials (circles, left *y*-axis) and logarithm of the experimental RRDE disc and ring currents (thin and thick crosses, respectively, right *y*-axis) versus the reaction descriptor *G*_HO_^80^. The numeric labels on red circles correspond to the different Sn structures in panel (**h**), while the other colored circles are labeled with the active metal within an MN_4_C motif (structure 4 in panel **h**). The solid colored and black lines represent ORR thermodynamic limits caused by the scaling relations for 2e^−^ and 4e^−^ pathways, respectively. The red vertical bands, obtained by crossing SnN_*x*_C log(*j*_disc_) with the thermodynamic volcano lines, highlight the *G*_HO_ values for which SnN_x_C would follow the same activity relationship as the other MNCs, i.e. for the pyridinic motifs number 2. **h** Different Sn-N-C motifs shown with adsorbed *OOH. Hydroxyl groups below the carbon matrix avoid fictitious electronic contributions from the site substrate.

Furthermore, the ex-situ low-temperature CO cryo-chemisorption and in-situ electrochemical nitrite adsorption followed by reductive stripping approaches have been developed to quantify the active sites and assess the intrinsic catalytic TOF of the M–N–C catalysts (Fig. 5a–f). Prior to probing the CO cryo-chemisorption, the thermal annealing pretreatment is required to remove any other adsorbates including oxygen^88^. This method (Fig. 5a, b) featured good specificity to M–N_*x*_ moieties due to the strong binding between CO and these single M–N_*x*_ sites, thus achieving reproducible active sites density (SD) values for different M–N–C catalysts. As expected, no detectable adsorption of CO was observed for metal-free nitrogen-doped carbon (N–C), while the amount of the adsorbed CO for the M–N–C catalysts followed the order: Fe–N–C > FeMn–N–C > Mn–N–C, which correlated well with the ORR activity^89^. In contrast, for the in-situ electrochemical adsorption/stripping approach, the ORR-active sites were blocked by the formed NO adducts on Fe–N–C catalysts and then five-electron electrochemical reductive stripping converted NO into ammonia (Fig. 5c). By the accurate determination of the stripping charge (*Q*_strip_, Fig. 5d) by subtracting the poisoned and unpoisoned traces in the stripping region, the active site density is obtained (Eq. (1):

1 $${{\rm {SD}}}\,\left[{{\rm {site}}}\,{{{\rm {nm}}}}^{-2}\right]=\frac{{Q}_{{{\rm {strip}}}}\left[C\,{g}^{-1}\right]\times {N}_{{\rm {A}}}\left[{{{\rm {mol}}}}^{-1}\right]}{{n}_{{{\rm {strip}}}}F\left[C\,{{{\rm {mol}}}}^{-1}\right]\times {{SA}}\left[{\rm {n{m}}}^{2}\,{{\rm {g}}}^{-1}\right]}$$

This method enables the researcher to obtain quantitative insights into the mass-based active site density under real ORR conditions^90^. The comparative study of the two methods above by Primbs et al. displayed remarkable quantitative congruence between the active SD and the TOF, and the measured active SD values ranged reproducibly on the same order of magnitude of 10^19^ sites g_catalyst_^−1^ despite being obtained from two vastly different analyses methods^86^. Moreover, the SD-TOF reactivity maps (Fig. 5e and f), derived from the ex-situ CO cryo-chemisorption technique and the in-situ nitrite probe technique, demonstrated that there are two routes for enhancing the ORR activity: (1) increasing the active SD and (2) improving the TOF of active sites.

To date, there appears to be a growing consensus that atomically dispersed Fe–N_4_ moieties possess the highest four-electron ORR activity among all the identified active sites in Fe–N–C catalysts due to their unique electronic properties^78^. Despite this, the combination of atomically dispersed Fe–N_4_ moieties and other active sites (e.g. NC or Fe_3_C@NC) was considered by some researchers to promote efficiently four-electron ORR with high onset potential in acidic media^91^. Besides, Mössbauer spectroscopy results demonstrated that atomically dispersed Fe–N_4_ moieties mainly included two types: ferrous low-spin Fe–N_4_ site (D1) and ferrous intermediate spin Fe–N_4_ site (D2). D1 mainly contributes to the high intrinsic ORR activity due to the high electron density around the Fe centers and the basicity of the co-existing NC, in particular for NH_3_-pyrolyzed Fe–N–C materials^84^. Moreover, in-situ XAS results showed the significant change of the local configuration of the Fe centers during the ORR process: from being located out of the N_4_-plane under reducing potential below the Fe^2+/3+^ redox potential to moving back in the N_4_-plane at high potential. This unusual phenomenon is related to the reversible Fe^2+/3+^ redox transitions, which optimized the binding strength of the Fe centers with the ORR intermediates^84^.

**Fig. 6:**
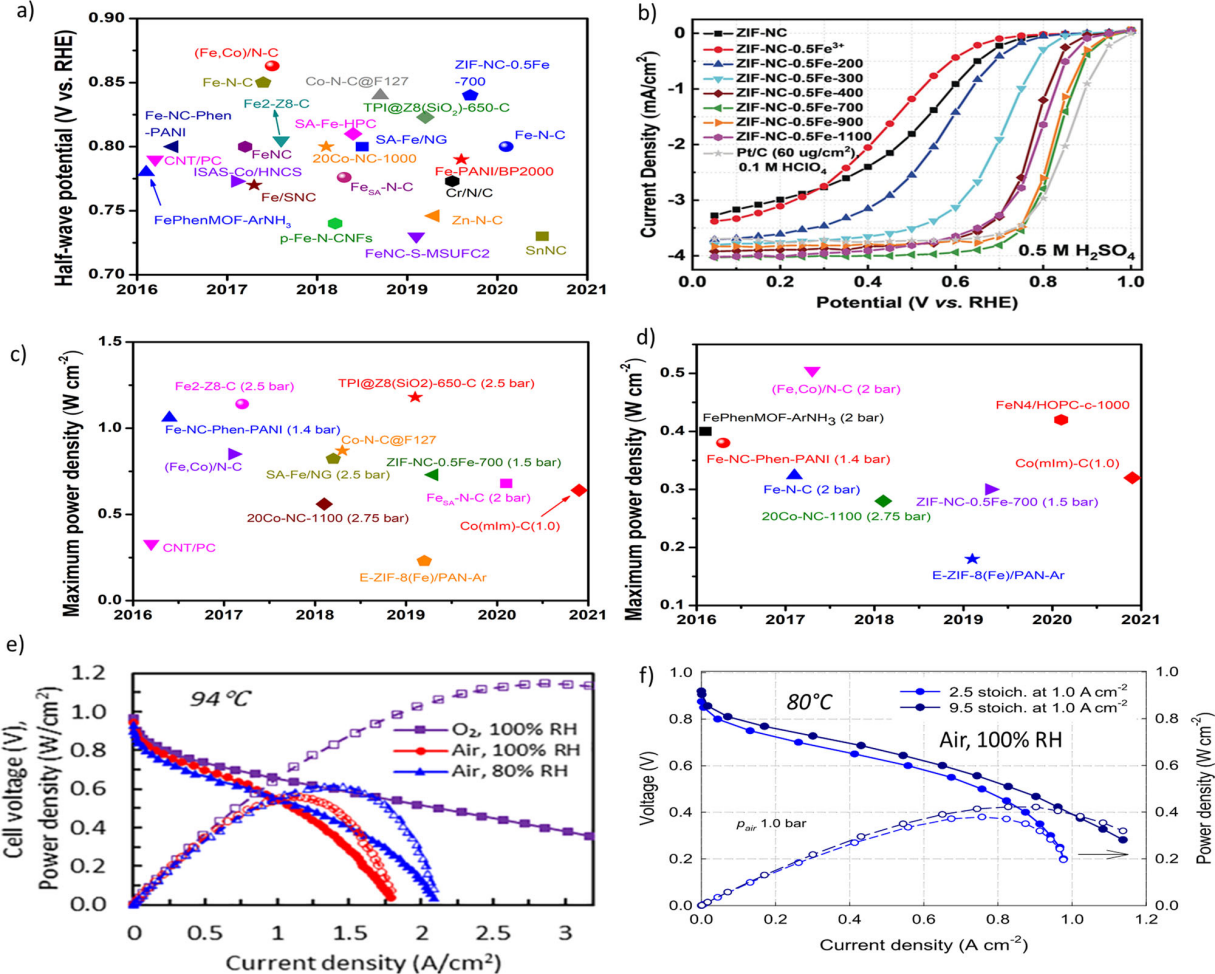
ORR performances of M–N–C catalysts in RRDE and PEMFCs. **a** Half-wave potential of various M–N–C catalysts tested in acidic electrolytes: FePhenMOF–ArNH_3_^84^, CNT/PC^93^, Fe–NC–Phen–PANI^15^, Fe/SNC^14^, ISAS–Co/HNCS^13^, FeNC^12^, Fe2–Z8–C^11^, Fe–N–C^10^, (Fe,Co)/N–C^97^, *p*-Fe–N–CNFs^9^, Fe_SA_–N–C^92^, Co–N–C^8^, SA-Fe/NG^7^, SA-Fe–HPC^6^, Co–N–C@F127^105^, FeNC–S–MSUFC2^95^, Zn–N–C^106^, Cr/N/C^107^, Fe-PANI/BP2000^5^, TPI@Z8(SiO_2_)–650–C^4^, ZIF–NC–0.5Fe–700^98^, Fe–N–C^3^, and SnNC^80^. **b** Steady-state ORR polarization curves showing the effect of activation temperature on the activity of ZIF–NC–0.5Fe^98^. **c** and **d** Maximum power density for PEMFCs using M–N–C as cathode catalysts under H_2_–O_2_ (**c**) and H_2_–air (**d**) conditions at 80 °C and 100% RH: **c** CNT/PC^93^, Fe–NC–Phen–PANI^15^, (Fe,Co)/N–C^97^, Fe2–Z8–C^11^, 20Co–NC-1100^8^, SA–Fe/NG^7^, Co–N–C@F127^105^, E-ZIF–8(Fe)/PAN–Ar^128^, ZIF–NC–0.5Fe–-700^98^, TPI@Z8SiO_2_-650-C^4^, Co(mIm)–NC(1.0)^108^, and Fe_SA_–N–C;^79^ **d** FePhenMOF–ArNH_3_^84^, Fe–NC–Phen–PANI^15^, Fe–N–C^129^, (Fe,Co)/N–C^97^, 20Co–NC-1100^8^, E-ZIF–8(Fe)/PAN–Ar^4^, ZIF-NC–0.5Fe-700^98^, Co(mIm)–NC(1.0)^108^, and FeN4/HOPC-c-1000^99^. Pressures are 1.0 bar unless specified otherwise. **e** Polarization and power density curves obtained under air and O_2_ for MEAs prepared from a chemically doped MOF-derived Fe−N−C catalyst. Conditions: 4.0 mg cm^-2^ loading, I/C of 0.6, Nafion 211 membrane, cell temperature: 94 °C; flow rate H_2_/air or O_2_: 200/1000 sccm, 1.7 atm H_2_/air or O_2_ partial pressure^130^. **f** H_2_–air fuel cell polarization plots. Cathode: ~4.0 mg cm^−2^ of (CM + PANI)–Fe–C; air 200 ml min^−1^ (2.5 stoichiometry at 1.0 A cm^−2^) and 760 ml min^−1^ (9.5 stoichiometry at 1.0 A cm^−2^); 100% relative humidity (RH); and 1.0 bar partial pressure. Anode: 2.0 mg_Pt_ cm^−2^ Pt/C; H_2_ 200 ml min^−1^; 100% RH; and 1.0 bar partial pressure. Membrane Nafion 211, cell 80 °C, electrode area 5 cm^2^ ^12^. Panels **a** and **b** adapted from ref. ^2^. Panel **e** reprinted with permission from ref. ^130^. Copyright 2019 American Chemical Society.

In addition to the identification of ORR-active sites, strategies to improve the kinetic activity of atomically dispersed Fe–N_4_ moieties have also been actively pursued (Fig. 6a, b). Recently, single-atom catalysts (SACs) afford the opportunity to optimize the kinetic activity of atomically dispersed Fe–N_4_ moieties because of the maximum atom utilization efficiency and the most exposed well-defined active sites^92^. To this end, extensive effort has been devoted to the fabrication of Fe–N–C SACs. A silica-protective-layer-mediated pyrolysis method has been proposed for the preferential formation of catalytically active Fe–N_4_ moieties in Fe–N–C SACs by efficiently preventing the formation and agglomeration of inorganic Fe-based NPs during the pyrolysis process^79,93^. Moreover, the formed porous structures upon removal of silica help in exposing more active sites and promoting mass transport. In addition, given the fact that pyrrolic nitrogen has been theoretically demonstrated to tune the O_2_ adsorption energy of the coordinated Fe atoms and activate the neighboring carbon atoms as ORR-active sites, high-purity pyrrole-type Fe–N_4_ catalyst has been successfully obtained through direct pyrolysis of polyaniline precursor under ammonia atmosphere^94^. An overview of the achievements in terms of maximum power density in PEMFC measurements employing M–N–C catalysts is shown in Fig. 6c–f.

Recent researches demonstrated that the kinetic activity of atomically dispersed Fe–N_4_ moieties is also influenced by the size of the carbon plane^95^. A large carbon plane causes the decreased ORR activity due to the strong adsorption of the Fe–N_4_ moieties for the ORR intermediates induced by highly delocalized electron-rich π-band. Alternatively, a small carbon plane also leads to the increase of carbon edge sites and thus brings stability issues due to their easy oxidation. In this regard, it is very challenging to control the proper size of the carbon plane without affecting the formation of Fe–N_4_ moieties. Edge nitrogen-modified divacancies (ND) trapped atomic Fe motifs (e-ND−Fe) were demonstrated theoretically to be more active than an intact center model (c-ND−Fe) due to the lower free-energy barrier resulting from the local electronic redistribution and bandgap shrinkage^96^. Besides, doping heteroatom (e.g. S, P) into Fe–N–C SACs has been reported to regulate the electronic structures of the Fe centers through introducing different coordination configurations due to the electron-withdrawing feature of heteroatom^77,95^. Meanwhile, N-coordinated Fe–Co dual sites embedded into N-doped carbon matrixs have been constructed by a host–guest strategy^97^.

Since the ORR process occurs in the triple-phase boundary, optimizing the porous structure of the catalysts is important for improving the utilization of the active sites and facilitating the mass transport of ORR-related species (O_2_, H_2_O, and proton) through the CL. Recently, zinc-based ZIFs (ZIF-8) has been widely used as carbon and nitrogen source for hosting the Fe–N_4_ moieties due to high nitrogen content and superior microporosity^4,98–100^. The formation of atomically dispersed active Fe–N_4_ moieties were found to originate from ultrafine FeO_*x*_ particles embedded into NC at the low temperature of 400 °C^98^. Nevertheless, the microporous feature of ZIF-derived Fe–N–C is adverse to efficient mass transport of the reactants and products, and the accessibility of active sites, which is further exacerbated when increasing the cathode layer thickness in practical PEMFCs. In contrast, macroporous and/or mesoporous structures were demonstrated to facilitate the efficient mass transport of the reactants and products toward and away from the active sites^99^. Based on this point, Jaouen’s group reported three-dimensional (3D) architecture of Fe–N–C catalysts with hierarchical micro-, meso-, and macro-porosity by co-electrospinning of Fe-doped ZIF-8 and polyacrylonitrile^4^.

Besides, the stability issues of these Fe–N–C catalysts remain a challenge for their practical application in PEMFCs^101^. To date, four kinds of instability mechanisms have been proposed, including the demetalation and the protonation of the active sites, H_2_O_2_ and/or free radicals induced surface oxidation, and micropore flooding^102,103^. Single-atom mobility and agglomeration into clusters should be also considered^104^. However, a broad consensus has been reached that the performance loss is most likely from the produced H_2_O_2_ during the ORR process, which could react with Fe–N_*x*_ moieties as a Fenton reagent’s catalyst to form reactive oxygen-containing free radicals^101^. These radicals could cause the oxidation degradation of proton exchange membrane employed in MEA-based FCs^105^, and especially nitrogen-doped carbon matrixs, further leading to Fe leaching and increased hydrophilicity with consequent micropore flooding. To solve this conundrum, extensive efforts have been devoted to the development of Fe-free M–N–C catalysts (M = Co, Zn, Cr, and Sn) possessing less Fenton-reaction activity^80,105–109^. For instance, a surfactant-assisted approach has been developed to fabricate highly active atomically dispersed Co-doped carbon catalyst^105^. The unique confinement effect from the cohesive interactions between the selected surfactant and Co-doped ZIF-8 was conducive to efficiently prevent the agglomeration of Co atomic sites. Besides Co–N–C catalyst, another specific family of Mn–N–C catalysts with atomically dispersed Mn–N_4_ sites has also been investigated^110^. Recently, our group reported p-block Sn–N–C catalyst with the catalytically active single-metal Sn–N_*x*_ moieties, exhibiting comparable ORR activity to the Fe–N–C catalyst in acidic media in terms of intrinsic catalytic TOF and fuel cell power density^80^. Particularly, after NH_3_ treatment, the Sn–N–C–NH_3_ catalysts exhibited a 40–50% higher current density than Fe–NC–NH_3_ at cell voltages below 0.7 V. Theoretical calculations demonstrated that the formed Sn–N_*x*_ moieties do not follow the usual linear relationships between the chemisorption strength of different ORR intermediates.

Generally, higher loading of PGM-free catalysts is needed to achieve comparable performance compared to PGM-based catalysts owing to the lower intrinsic ORR activity, causing order of magnitude thicker CL than PGM-based catalysts^111^. The thicker CL cannot make effective use of the catalyst and also increase the transport resistance of the reactants and products. The optimization of CL structure at micro-/nano-scale has shown very importantly to regulate the transport properties. Generally, there are many factors influencing the CL structure, such as pore-size distribution of catalyst, ionomer loading and distribution, and the deposition process^112^. The pore-size distribution of catalyst and the ionomer loading can affect the degree of ionomer infiltration into the catalyst and the ionomer distribution, further influencing the local microstructural transport resistance and the ionic conductivity within the CL. Besides, the produced water during the PEMFC operation can block the gas-accessible active sites or inhibit mass transport when pooling at the interfaces^111^. Optimizing the CL’s wettability is an effective strategy to alter the water accumulation and transport along with avoiding the blockage of pores within the CL. In addition, the non-uniform drying process can also cause the formation of large cracks and voids at component interfaces, resulting in increased tortuosity^111^. In this regard, some novel approaches, including layered electrodes with a gradient of ionomer from the membrane to gas diffusion layer^1^, have been developed to balance the tradeoff between gas transport resistance and proton transport resistance of the bulk electrode.

To better understand the morphologic structures and transport processes of CL, some ex-situ techniques have been explored, including mercury intrusion porosimetry^113^, focused ion beam scanning electron microscope (FIB-SEM) or scanning transmission electron microscope (STEM)^114^. Although high-resolution 3D morphology of CL can be obtained through FIB-SEM and STEM, there are some challenges for these methods, for instance, FIB-SEM itself is a destructive technique. Alternatively, micro-/nano-scale resolution X-ray computed tomography (CT) provides a feasible non-destructive operando method for visualizing the morphologic structure, water, and ionomer distributions of CL^111^.

## Future directions and perspectives

To accelerate the widespread commercialization of PEMFCs, two kinds of cathode catalyst systems have been mainly explored, including nanostructured PGM-based materials with low-usage of Pt by alloying mostly with 3*d* transition metals (i.e. Ni, Co, Fe, Cu) or rare earths (i.e. Sc, Y, La, Ce, Gd, Tb), and PGM-free M–N–C (M = Fe, Co, Zn, Mn, Cr, and Sn), and engineering the triple-phase boundary at the cathode layer through regulating the surface properties of carbon supports and ionomer/catalyst interactions. Despite great advances that have been achieved, there remain some challenges for the further improvement of their performance and the optimization of the cathode layers. Further work in this field should continue to concentrate on the following aspects.

### Improvement in stability and MEA-performance of nanostructured PGM-based catalysts

Further development of PGM-based catalysts aimed at their implementation in low-Pt cathodes in PEMFCs should focus on improving the stability and achieving high MA in MEA-based FCs measurements, resembling the enhancement factors reported in RDE measurements. To this end, the carbon support, and more generally the triple-phase boundary at the active site, should be considered in the design. This will involve tuning the carbon support porosity, optimizing particle location, applying surface chemical modifications to the carbon, and modifying the ionomer backbone. Ionomer poisoning of the active sites might indeed be a severe problem for the emerging nanostructured PGM-based catalysts, hindering the translation of their high activity. More specific tuning of the catalysts alone should keep their ECSA as high as possible to mitigate local oxygen resistances, as well as improve the chemical and morphological stability by translating the optimized MEA-preparation and activation protocols for Pt and pseudo-spherical Pt alloy to these advanced Pt-alloy nanostructures. This includes optimization of leaching and annealing treatments to prevent metal leaching during operations. Finally, we note that improvements should target specific applications. For example, for light-duty vehicles, high power densities are required while for heavy duty vehicles the requirements for high power densities can be lowered, considering their major focus on high efficiency operating at higher voltages and lower power densities (in addition to stability)^115^.

### Improvement in the catalytic activity and durability for PGM-free catalysts

Currently, insufficient intrinsic activity and density of catalytically active sites remain as the Achilles’ heel of PGM-free M–N–C SAC relative to PGM-based catalysts, which are critical factors for efficiently reducing the thickness of the cathode layer in PEMFCs and promoting the translation of PGM-free catalysts into high MEA performance. Besides, the long-term durability of PGM-free catalysts under MEA operating conditions is also a major challenge for commercialization and needs to be addressed. In this regard, some Fe-free M–N–C SACs, i.e. Sn–N–C, have been developed because of the low Fenton-reaction activity, in spite of the inferior activity compared to the Fe–N–C SACs. To improve the intrinsic activity, the design of PGM-free M–N–C SAC with dual single-atomic metal active sites to optimize the adsorption strengths of the active sites to the key ORR intermediates is one of the promising strategies. However, there are only a few reports in the literature to date. Meanwhile, from the viewpoint of the density of the active site, increasing the metal loadings within SACs is highly desired, but this has been largely hampered since the isolated metal atoms are easily aggregated owing to high surface energy during the high-temperature pyrolysis. Besides, hierarchical porous structures comprising micro-, meso-, and macropores are also desirable to improve catalytic performances. In these structures atomically dispersed M–N_*x*_ moieties, which act as active sites, are hosted within micropores, whereas meso-/macropores facilitate the mass transport of reactants and products. Based on the considerations above, there is a continuous incentive to search for novel efficient and facile synthetic strategies for the rational construction of SACs with multiple single-atomic metal sites, high metal loadings, and rich porous structures. It is worth stressing that testing of activity under fuel cell operating conditions needs to be done in a differential cell to avoid pressure drop across the cathode that directly translates into higher O_2_ activity, making results unreliable.

### Optimization in the cathode layer design in the MEA test

The surface properties and porous structure of the carbon support have also significant influences on the output power density and stability of the PEMFCs. On one hand, the stable attachment and exposure of active sites are closely related to the surface properties and pore structure of carbon support. On the other hand, the configuration and distribution of the ionomer used for MEA fabrication can be tuned by optimizing the surface properties and pore structure of carbon support, the configuration, and structure of the ionomer itself, further reducing the local oxygen diffusion resistance at the triple-phase boundary. Meanwhile, the corrosion resistance of carbon support can be enhanced by improving the graphitization degree, thus efficiently mitigating the collapse of the triple-phase boundary, the risk of water flooding, and the demetalation of active sites. Despite several successful examples for optimizing cathode layer have been achieved, more MEA-specific in-situ characterization techniques need to be developed to establish the influence mechanism of the cathode layer components under working conditions of PEMFCs.

## Supplementary information

Supplementary Information
