# Supplementary material for: Advancements in cathode catalyst and cathode layer design for proton exchange membrane fuel cells
Source: Nat Commun. 2021 Oct 13;12:5984. doi: 10.1038/s41467-021-25911-x (PMC8514433; doi:10.1038/s41467-021-25911-x)
Supplement: Supplementary file 1 — Supplementary Information [file 41467_2021_25911_MOESM1_ESM.pdf]

Advancements in Cathode Catalyst and Cathode Layer Design for Proton Exchange Membrane Fuel Cells

Sun et al.

Supplementary Table 1 Comparison of electrochemical ORR performance for selected PGM-based catalysts from Figure 3c, d.

Catalyst	RDE loading (μg_{Pt} cm^{-2})	RDE-MA (A mg_{Pt}^{-1})	FC cathode loading (mg_{Pt} cm^{-2})	FC MA @ 0.9 V (A mg_{Pt}^{-1})	FC PD @ 0.6 V (W cm^{-2})
Pt_xY/C[1]	8.7	0.106	0.12	0.101	0.48
PtNi NWs[2]	--	--	0.1	0.577	~0.46*
PtCo/HSC[3, 4]	--	--	0.1	0.6	1.05*
$L1_0$-CoPt[5]	~8.08	2.26	0.105	0.56	0.57*(0.66* at RH20%)
$L1_0$-CoPt/NMGC-1[6]	--	--	--	0.91*	--
PtNi nanopinwheels[7]	--	--	0.03	0.5	--
50% PtNi/C[8]	--	--	0.1 (0.2)	0.89 (0.44)	--
PtNi/C [9]	--	--	0.2	~0.34*	1.008*
LP@PF-1[10]	3.0	8.64	0.033	1.08	0.72
LP@PF-2[10]	3.2	12.36	0.035	1.77	0.78
Pt_3Co-ZIF [10]	3.9	~3.2*	0.043	0.417	--
oh-PtNi(Mo)[11]	10	3.43	0.1	0.45	0.702
PtCo/CCCS-800[12]	--	--	0.1	0.39	--
3M UTF PtNiIr[13]	--	--	Total loading<0.09	0.44	--
3M UTF[14]	--	--	0.025	0.31	--
3M NPTF[14]	--	--	0.12	0.47	--
Meso-PtNi-2[15]	--	--	0.11	1.34	--

Note: RDE: rotating disk electrode; MA: mass activity; FC: fuel cell; PD: power density. RH: relative humidity. *: values estimated by us from the published graphs. For further details and conditions see the caption of Figure 3.

Supplementary Table 2 Comparison of electrochemical ORR performance for selected PGM-free catalysts in RDE and MEA-based fuel cells.

Catalyst	Loading ($mg\ cm^{-2}$)	$E_{1/2}$ (V)	MA ($A\ g^{-1}$)	PPD in H_2-air ($W\ cm^{-2}$)	PPD in H_2-O_2 ($W\ cm^{-2}$)
FePhenMOF –$ArNH_3$[16]	0.6 @ RDE<br>2.0 @PEMFC	0.78	7.8 @0.8 V	0.4 @0.4 V	-
CNT/PC[17]	0.8 @ RDE<br>3.05 @PEMFC	0.79	5.6 @0.8 V	-	0.58 @0.6 V
Fe-NC-Phen-PANI[18]	0.6 @ RDE<br>4.0 @PEMFC	0.80	-	0.38 @0.56 V	1.06 @0.46 V
Fe/SNC[19]	0.6 @ RDE	0.77	-	-	-
ISAS-Co/HNCS[20]	0.501 @ RDE	0.773	-	-	-
FeNC[21]	0.6 @ RDE	0.8	-	-	-
Fe2-Z8-C[22]	0.4 @ RDE<br>2.8 @PEMFC	0.805	-	-	1.141
Fe-N-C[23]	0.8 @ RDE	0.85	-	-	-
(Fe,Co)/N-C[24]	1.095 @ RDE<br>0.77 @PEMFC	0.863	2.6 @0.9 V	0.505 @0.42 V	0.98 @0.85 V
p-Fe-N-CNFs[25]	0.6 @ RDE	0.74	11.5 @0.74 V	-	-
Fe_{SA}-N-C[26]	0.28 @ RDE	0.776	34.3 @0.75 V	-	-
Co-N-C[27]	0.8 @ RDE<br>4.0 @PEMFC	0.8	-	0.28	0.56
SA-Fe/NG[28]	0.6 @ RDE<br>2.0 @PEMFC	0.8	26 @0.75 V	-	0.823
SA-Fe-HPC[29]	0.2 @ RDE	0.81	-	-	-
Co-N-C@F127[30]	0.8 @ RDE<br>4.0 @PEMFC	0.84	-	0.28	0.87
FeNC-S-MSUFC2[31]	0.815 @ RDE	0.73	-	-	-
Zn-N-C[32]	0.5 @ RDE	0.746	-	-	-
Cr/N/C[33]	0.6 @ RDE	0.773	-	-	-
Fe-PANI /BP2000[34]	0.6 @ RDE	0.79	0.592 @0.8 V	-	-
TPI@Z8 (SiO_2)-650-C[35]	0.4 @ RRDE<br>2.0 @PEMFC	0.823	6.7 @0.8 V	0.42	1.18 @0.47 V
ZIF-NC-0.5Fe-700[36]	0.6 @ RDE<br>3.5 @PEMFC	0.84	-	0.32	0.73
Fe-N-C[37]	1.0 @ RDE	0.70	-	-	-
SnNC[38]	0.8 @ RRDE	0.73	-	-	-

Fe-N-C[39]	-	-	-	0.324	0.49
E-ZIF-8 (Fe)/PAN-Ar[40]	0.8 @ RDE<br>4.0 @PEMFC	-	0.125 @0.85 V	0.18	0.23
Co(mIm)–NC(1.0)[41]	0.6 @ RRDE<br>5.8 @PEMFC	0.82	-	0.32	0.64
Fe_{SA}-N-C[42]	0.28 @ RDE<br>3.0 @PEMFC	0.8	6.14 @0.8 V	-	0.68
FeN_4/HOPC-c-1000[43]	4.0 @PEMFC	0.8	-	-	0.69

Note: $E_{1/2}$: Half-wave potential; MA: mass activity in RDE; PPD: peak power density in PEMFC;

Supplementary References

1. Schwammlein JN, Harzer GS, Pfandner P, Blankenship A, El-Sayed HA, Gasteiger HA. Activity and Stability of Carbon Supported PtxY Alloys for the ORR Determined by RDE and Single-Cell PEMFC Measurements. *J Electrochem Soc* **165**, J3173-J3185 (2018).
2. Alia S, Pivovar BS. Extended Surface Electrocatalyst Development. In: *DOE Hydrogen and Fuel Cells Program 2018 Annual Merit Review and Peer Evaluation Meeting* (ed Energy USDo) (2018).
3. Kongkanand A, Mathias MF. The Priority and Challenge of High-Power Performance of Low-Platinum Proton-Exchange Membrane Fuel Cells. *J Phys Chem Lett* **7**, 1127-1137 (2016).
4. Yarlagadda V*, et al.* Boosting Fuel Cell Performance with Accessible Carbon Mesopores. *Acs Energy Lett* **3**, 618-621 (2018).
5. Li J*, et al.* Hard-Magnet L10-CoPt Nanoparticles Advance Fuel Cell Catalysis. *Joule* **3**, 124-135 (2019).
6. Spendelow JS. Advanced Electro-Catalysts through Crystallographic Enhancement. In: *DOE Hydrogen and Fuel Cells Program 2020 Annual Merit Review* (ed Energy USDo) (2020).
7. Stamenkovic V, Markovic NM. Tailored high performance low-PGM alloy cathode catalysts In: *2018 DOE Hydrogen and Fuel Cells Program Review* (ed Energy USDo) (2018).
8. Dionigi F*, et al.* D4.3:DEMONSTRATION OF A CATALYTIC ENTITY SHOWING 0.7A/mgPt, IN AN MEA TEST AND A SURFACE AREA >40m2/gPt AFTER 30,000 CYCLES FROM 0.6 TO 0.925V, https://www.gaia-fuelcell.eu/images/D4.3_Catalytic_activity_and_stability_in_MEA.pdf (2020).
9. Ercolano Gea. DELIVERABLE D3.2 – CATALYST ACTIVITY AND STABILITY STUDIES (2017), https://www.inspire-fuelcell.eu/images/INSPIRE_D3.2_Catalyst_activity_and_stability_PUBLIC_version_final.pdf (2017).
10. Chong L*, et al.* Ultralow-loading platinum-cobalt fuel cell catalysts derived from imidazolate frameworks. *Science* **362**, 1276-1281 (2018).

11. Dionigi F*, et al.* Controlling Near-Surface Ni Composition in Octahedral PtNi(Mo) Nanoparticles by Mo Doping for a Highly Active Oxygen Reduction Reaction Catalyst. *Nano letters* **19**, 6876-6885 (2019).
12. Jung WS, Popov BN. New Method to Synthesize Highly Active and Durable Chemically Ordered fct-PtCo Cathode Catalyst for PEMFCs. *ACS Appl Mater Interfaces* **9**, 23679-23686 (2017).
13. Steinbach AJ. Highly active, durable, and ultra-low PGM NSTF thin film ORR catalysts and supports (ed Energy USDo) (2017).
14. Steinbach AJ. Highly Active, Durable, and Ultra-low PGM NSTF Thin Film ORR Catalysts and Supports. (ed Energy USDo) (2016).
15. Kim HY*, et al.* Self-supported mesostructured Pt-based bimetallic nanospheres containing an intermetallic phase as ultrastable oxygen reduction electrocatalysts. *Small* **12**, 5347-5353 (2016).
16. Li J*, et al.* Structural and mechanistic basis for the high activity of Fe–N–C catalysts toward oxygen reduction. *Energy & Environmental Science* **9**, 2418-2432 (2016).
17. Sa YJ*, et al.* A General Approach to Preferential Formation of Active Fe-Nx Sites in Fe-N/C Electrocatalysts for Efficient Oxygen Reduction Reaction. *Journal of the American Chemical Society* **138**, 15046-15056 (2016).
18. Fu X*, et al.* In Situ Polymer Graphenization Ingrained with Nanoporosity in a Nitrogenous Electrocatalyst Boosting the Performance of Polymer-Electrolyte-Membrane Fuel Cells. *Adv Mater* **29**, (2017).
19. Shen H*, et al.* Synergistic Effects between Atomically Dispersed Fe-N-C and C-S-C for the Oxygen Reduction Reaction in Acidic Media. *Angew Chem Int Ed Engl* **56**, 13800-13804 (2017).
20. Han Y*, et al.* Hollow N-Doped Carbon Spheres with Isolated Cobalt Single Atomic Sites: Superior Electrocatalysts for Oxygen Reduction. *Journal of the American Chemical Society* **139**, 17269-17272 (2017).
21. Chung HT*, et al.* Direct atomic-level insight into the active sites of a high-performance PGM-free ORR catalyst. *Science* **357**, 479-483 (2017).
22. Liu Q, Liu X, Zheng L, Shui J. The Solid-Phase Synthesis of an Fe-N-C Electrocatalyst for High-Power Proton-Exchange Membrane Fuel Cells. *Angew Chem Int Ed Engl* **57**, 1204-1208 (2018).
23. Zhang H*, et al.* Single Atomic Iron Catalysts for Oxygen Reduction in Acidic Media: Particle Size Control and Thermal Activation. *Journal of the American Chemical Society* **139**, 14143-14149 (2017).
24. Wang J*, et al.* Design of N-Coordinated Dual-Metal Sites: A Stable and Active Pt-Free Catalyst for Acidic Oxygen Reduction Reaction. *Journal of the American Chemical Society* **139**, 17281-17284 (2017).
25. Hu B-C*, et al.* SiO_2-protected shell mediated templating synthesis of Fe–N-doped carbon nanofibers and their enhanced oxygen reduction reaction performance. *Energy & Environmental Science* **11**, 2208-2215 (2018).
26. Jiao L, Wan G, Zhang R, Zhou H, Yu SH, Jiang HL. From Metal-Organic Frameworks to Single-Atom Fe Implanted N-doped Porous Carbons: Efficient Oxygen Reduction in Both Alkaline and Acidic Media. *Angew Chem Int Ed Engl* **57**, 8525-8529 (2018).
27. Wang XX*, et al.* Nitrogen-Coordinated Single Cobalt Atom Catalysts for Oxygen Reduction in Proton Exchange Membrane Fuel Cells. *Adv Mater* **30**, (2018).

28. Yang L*, et al.* Unveiling the high-activity origin of single-atom iron catalysts for oxygen reduction reaction. *Proceedings of the National Academy of Sciences of the United States of America* **115**, 6626-6631 (2018).
29. Zhang Z, Sun J, Wang F, Dai L. Efficient Oxygen Reduction Reaction (ORR) Catalysts Based on Single Iron Atoms Dispersed on a Hierarchically Structured Porous Carbon Framework. *Angew Chem Int Ed Engl* **57**, 9038-9043 (2018).
30. He Y*, et al.* Highly active atomically dispersed CoN_4 fuel cell cathode catalysts derived from surfactant-assisted MOFs: carbon-shell confinement strategy. *Energy & Environmental Science* **12**, 250-260 (2019).
31. Mun Y, Lee S, Kim K, Kim S, Han JW, Lee J. Versatile Strategy for Tuning ORR Activity of a Single $Fe-N_4$ Site by Controlling Electron-Withdrawing/Donating Properties of a Carbon Plane. *Journal of the American Chemical Society* **141**, 6254-6262 (2019).
32. Li J*, et al.* Ultrahigh-Loading Zinc Single-Atom Catalyst for Highly Efficient Oxygen Reduction in Both Acidic and Alkaline Media. *Angew Chem Int Ed Engl* **58**, 7035-7039 (2019).
33. Luo E*, et al.* Single-Atom $Cr-N_4$ Sites Designed for Durable Oxygen Reduction Catalysis in Acid Media. *Angew Chem Int Ed Engl* **58**, 12469-12475 (2019).
34. Chen MX*, et al.* Identification of Catalytic Sites for Oxygen Reduction in Metal/Nitrogen-Doped Carbons with Encapsulated Metal Nanoparticles. *Angew Chem Int Ed Engl* **59**, 1627-1633 (2020).
35. Wan X*, et al.* Fe–N–C electrocatalyst with dense active sites and efficient mass transport for high-performance proton exchange membrane fuel cells. *Nat Catal* **2**, 259-268 (2019).
36. Li J*, et al.* Thermally Driven Structure and Performance Evolution of Atomically Dispersed FeN_4 Sites for Oxygen Reduction. *Angew Chem Int Ed Engl* **58**, 18971-18980 (2019).
37. Al-Zoubi T*, et al.* Preparation of Nonprecious Metal Electrocatalysts for the Reduction of Oxygen Using a Low-Temperature Sacrificial Metal. *Journal of the American Chemical Society* **142**, 5477-5481 (2020).
38. Luo F*, et al.* P-block single-metal-site tin/nitrogen-doped carbon fuel cell cathode catalyst for oxygen reduction reaction. *Nat Mater*, (2020).
39. Workman MJ, Serov A, Tsui L-k, Atanassov P, Artyushkova K. Fe–N–C Catalyst Graphitic Layer Structure and Fuel Cell Performance. *Acs Energy Lett* **2**, 1489-1493 (2017).
40. Li J*, et al.* Designing the 3D Architecture of PGM-Free Cathodes for H_2/Air Proton Exchange Membrane Fuel Cells. *ACS Applied Energy Materials* **2**, 7211-7222 (2019).
41. Xie XH*, et al.* Performance enhancement and degradation mechanism identification of a single-atom Co-N-C catalyst for proton exchange membrane fuel cells. *Nat Catal* **3**, 1044-1054 (2020).
42. Jiao L*, et al.* Nanocasting SiO_2 into metal-organic frameworks imparts dual protection to high-loading Fe single-atom electrocatalysts. *Nat Commun* **11**, 2831 (2020).
43. Qiao M*, et al.* Hierarchically Ordered Porous Carbon with Atomically Dispersed FeN_4 for Ultraefficient Oxygen Reduction Reaction in Proton-Exchange Membrane Fuel Cells. *Angew Chem Int Ed Engl* **59**, 2688-2694 (2020).
